# SCFA Alterations in IBS Subtypes: Systematic Evidence and Future Prospects for Biomarker-Based Diagnosis in India

**DOI:** 10.4014/jmb.2605.05029

**Published:** 2026-09-02

**Authors:** Sanika Limaye, Prajkta Bhide, Anup Kale, Neha Bokey

**Affiliations:** 1 Department of Biosciences and Technology, Dr. Vishwanath Karad MIT World Peace University, Pune, India; 2 Department of Public Health, Dr. Vishwanath Karad MIT World Peace University, Pune, India

**Keywords:** Irritable bowel syndrome (IBS), Short-chain fatty acids (SCFA), Acetate, Butyrate, Propionate, Systematic review, Biomarkers

## Abstract

Irritable bowel syndrome (IBS) is a chronic disorder of gut–brain interaction characterized by abdominal pain and altered bowel habits. Short-chain fatty acids (SCFAs), microbial metabolites of dietary fiber, play key roles in gut physiology and have been implicated in IBS pathogenesis. Propionate, acetate, and butyrate are of particular interest as potential biomarkers for diagnosis and subtype differentiation. A systematic search of PubMed, Web of Science, and Embase identified studies reporting fecal SCFA concentrations in IBS patients versus healthy controls (HCs). Adult cohorts diagnosed by Rome criteria, case–control or randomized designs, and sufficient SCFA data for effect size calculation were included. Data were harmonized to mean ± SD and analyzed; pooled standardized mean differences (SMDs) with 95% confidence intervals (CI) were calculated, with subgroup analyses for IBS-D and IBS-C. Methodological quality was assessed using the Newcastle–Ottawa Scale (NOS). Eleven studies met inclusion criteria. Across all IBS cohorts, propionate was significantly elevated compared to HCs, while acetate and butyrate showed no differences. In IBS-D, propionate was consistently higher. In IBS-C, total SCFAs were significantly reduced, with negligible heterogeneity. Acetate, propionate, and butyrate remained non-significant, with moderate to substantial heterogeneity. NOS assessment indicated that most studies were of good quality, with one rated moderate. This meta-analysis highlights propionate elevation as a biomarker for IBS, particularly IBS-D, and reduced total SCFAs as a distinct metabolic signature in IBS-C. Variability in individual metabolites reflects dietary and methodological influences. The absence of Indian cohorts limits generalizability; future studies integrating SCFA profiling with microbiome analysis in Indian populations are needed to validate biomarker utility and support precision management of IBS.

## Background

Irritable bowel syndrome (IBS) is a chronic disorder of gut–brain interaction defined by recurrent abdominal pain associated with altered bowel habits, in the absence of structural or biochemical abnormalities. Diagnosis is based on symptom-based criteria, most widely the Rome IV framework, which emphasizes abdominal pain related to defecation and changes in stool frequency or form [1]. Globally, IBS affects approximately 7–10% of the population, though prevalence varies by geography, diagnostic criteria, and cultural reporting patterns [2]. Subtypes include diarrhea-predominant (IBS-D), constipation-predominant (IBS-C), mixed (IBS-M), and unclassified (IBS-U), each with distinct symptom burden and therapeutic response profiles [1].

Short-chain fatty acids (SCFAs), primarily acetate, propionate, and butyrate, are microbial metabolites generated by fermentation of dietary fiber. They exert diverse physiological effects, including modulation of intestinal motility, maintenance of epithelial barrier integrity, and regulation of immune responses [3]. Altered SCFA profiles have been implicated in IBS pathogenesis, with propionate linked to visceral hypersensitivity and dysmotility, and butyrate associated with epithelial health and anti-inflammatory effects [4]. Thus, SCFA analysis provides a promising avenue for identifying metabolic biomarkers that reflect microbiota–host interactions in IBS.

The burden of IBS in India is considerable, with prevalence estimates ranging from 4–7% among adults, though underdiagnosis remains common due to cultural perceptions and limited access to gastroenterology services [5]. Subtype distribution shows IBS-D predominance in urban populations, likely linked to dietary transitions and stress, while IBS-C is more frequent in rural cohorts, reflecting higher fiber intake and lifestyle differences [6]. Given India’s dietary diversity and unique microbiome signatures, SCFA profiling could provide valuable insights into regional IBS phenotypes and guide personalized management strategies.

Given these considerations, profiling SCFA concentrations holds significant clinical relevance for both the diagnosis and therapeutic management of IBS. Biomarker-based approaches could complement symptom-based Rome IV criteria, reduce misclassification, and enable targeted dietary or microbiome-modulating interventions. In India, where functional gastrointestinal disorders are rising with urbanization, SCFA biomarkers such as propionate may help stratify patients by subtype and improve therapeutic precision. This systematic review and meta-analysis aimed to synthesize global evidence on fecal SCFA concentrations in IBS and its subtypes, evaluate their potential as biomarkers, and highlight implications for Indian cohorts, where such data remain scarce.

## Methodology

### Information Sources and Search Strategy

A systematic literature search was conducted across PubMed, Web of Science, and Embase for all published articles from 2000-2025. Search terms included “irritable bowel syndrome”, “IBS”, “short-chain fatty acids”, “SCFAs”, “acetate”, “propionate”, “butyrate”. Boolean operators (AND, OR, NOT) were applied to refine results. Review articles, meeting abstracts, non-English publications, and animal studies were excluded. Reference lists of selected articles were also screened to identify additional eligible studies.

### Study Selection and Eligibility Criteria

Titles and abstracts of retrieved records were screened for relevance, followed by full-text evaluation according to predefined inclusion and exclusion criteria. Inclusion criteria were: (1) IBS diagnosis based on Rome criteria (or described as IBS prior to Rome I, 1990); (2) case–control design for IBS patients vs. healthy controls (HCs), or randomized/self-controlled design for treatment comparisons; (3) availability of fecal SCFA data sufficient to calculate mean difference (MD) or standardized mean difference (SMD) with 95% confidence intervals (CI); (4) adult IBS patients and HCs comparable in age and sex. Exclusion criteria were: (1) IBS patients with other gastrointestinal diseases; (2) animal studies; (3) lack of full text or insufficient data for effect size calculation.

### Data Extraction

Extracted information included: (1) first author and publication year; (2) country; (3) IBS subtype; (4) diagnostic criteria; (5) participant demographics (age, sex, number); (6) fecal SCFA data format; and (7) analytical methods used for fecal SCFA measurement. Discrepancies were resolved through discussion until consensus was achieved.

### Quality Assessment

Methodological quality of included 11 studies was assessed using the Newcastle–Ottawa Scale (NOS). The NOS, designed for non-randomized observational studies, was applied uniformly across all included studies for consistency. One study (Halmos 2015) was a randomized-controlled trial (RCT), but its inclusion under NOS did not materially affect the overall quality assessment. Each study was evaluated across three domains (Selection, Comparability, Outcome/Exposure). Studies were scored within each domain according to NOS criteria (maximum 4 for Selection, 2 for Comparability, 3 for Outcome/Exposure). For visualization, scores were converted into categorical ratings: ‘Low risk’ (≥3 score in Selection, 2 in Comparability, ≥2 in Outcome), ‘Some concerns’ (2 score in Selection, 1 in Comparability, 1 in Outcome), and ‘High risk’ (≤1 score in Selection, 0 in Comparability, ≤1 in Outcome). A traffic-light plot was generated in R (4.5.2) using the ‘ggplot2’ and ‘reshape2’ packages to visualize the distribution of scores across domains. A total score of ≥6 indicated good research quality (Table 2, supplementary material). Publication bias was planned to be assessed using funnel plots and Egger’s regression when ≥10 studies were available in a pooled analysis. As all subgroups in this review included fewer than 10 studies, formal assessment of publication bias was not feasible.

### Statistical Analysis

Meta-analysis was conducted in R (version 4.5.2) using the ‘meta’ and ‘metafor’ packages for statistical calculations. Forest plots were generated using MetaAnalysisOnline (www.metaanalysisonline.com, accessed January 2026). For continuous outcomes, MD values were calculated when units were consistent, and SMD values when units differed, both with 95% CI. Mean and standard deviation (SD) were estimated from median and interquartile ranges using Wan’s method, or derived from mean and CI following Cochrane Handbook recommendations. Heterogeneity was assessed using the Chi² test and I² statistic. An I² < 50% and *p* > 0.1 indicated low heterogeneity. Subgroup analyses were conducted by IBS subtype to explore sources of heterogeneity. Statistical significance was set at *p* < 0.05.

## Results

### Selection of Studies

On specific query/keyword search in the databases, 525 publications were retrieved. Of them, 439 were excluded for duplication. For the 86 remaining for abstract screening, 57 were excluded for meeting one or more exclusion criteria. 29 remaining articles were selected for full text reading of which 18 were again excluded for meeting one or more exclusion criteria. In the end, 11 studies (Tana *et al*. (2010)[7], Chassard *et al*.(2012)[8], Halmos *et al*. (2015)[9], Ringel Kulka *et al*. (2015)[10], Farup *et al*. (2016)[11], Tian *et al*. (2020)[12], Fukui *et al*. (2020)[13], Sagar *et al*.(2020)[14], Sun *et al*. (2021)[15], Waseem *et al*. (2023)[16] and Shin *et al*. (2024)[17]) qualified for the systematic review. The PRISMA flowchart (Fig. 1) illustrates the study selection process, with 11 studies included in the final synthesis.

### Study Characteristics

The selected studies ranged from year 2010 to 2024. The available data on fecal SCFAs from the 11 included studies were reported in various formats, most commonly as mean ± SD. In other studies, results were expressed as median with interquartile range (IQR), mean with 95% CI, mean ± standard error of the mean (SEM), or estimated marginal mean (EMM) ± SEM. For consistency in quantitative synthesis, all data reported in alternative formats were converted into mean ± SD values. The studies used either gas chromatography (GC) for fecal SCFA detection, or other techniques such as gas–liquid chromatography (GLC), gas chromatography–mass spectrometry (GC-MS), proton nuclear magnetic resonance (1H NMR) and high-performance liquid chromatography (HPLC) (Table 1, Supplementary material).

### Quality Assessment

Methodological quality assessment using the NOS is summarized in (Table 2 and Fig. 1, Supplementary material). The traffic-light plot (generated in R, version 4.5.2, using the ‘ggplot2’ and ‘reshape2’ packages) illustrates the distribution of ratings across the three NOS domains (Selection, Comparability, Outcome/Exposure) for the 11 included studies (Fig. 1, Supplementary material). Outcome assessment was consistently rated as low risk, reflecting standardized laboratory methods and clear diagnostic criteria. Selection domains were predominantly low risk, with only one study showing some concerns due to recruitment limitations. The comparability domain showed the greatest variability, with several studies rated as ‘Some concerns’ due to incomplete adjustment for confounding factors such as dietary intake, BMI, age, and sex. No study was judged to be at high risk in any domain.

### Analysis of Studies

A total of eleven studies were included in the meta-analysis, examining fecal concentrations of total SCFAs and individual metabolites (acetate, propionate, butyrate) in patients with IBS compared to HCs.

For total fecal SCFAs (Fig. 2A), three studies- Halmos 2014, Farup 2016, and Ringel Kulka 2015- contributed data, encompassing 166 IBS patients and 64 controls. The pooled SMD was 0.03 (95% CI –0.27 to 0.33), with negligible heterogeneity (I² = 0%), indicating no significant difference in overall fecal SCFA concentrations between IBS patients and HCs. For acetate (Fig. 2B), five studies-Tana 2010, Halmos 2014, Farup 2016, Fukui 2020, and Ringel Kulka 2015- were analyzed, including 277 IBS patients and 116 controls. The pooled SMD was 0.06 (95% CI –0.29 to 0.41), with moderate heterogeneity (I² = 51.7%). While some studies reported higher acetate in IBS patients, others showed reductions or no difference, resulting in an overall non-significant effect. For butyrate (Fig. 2C), four studies- Halmos 2014, Farup 2016, Fukui 2020, and Ringel Kulka 2015- were included, with 251 IBS patients and 90 controls. The pooled SMD was –0.08 (95% CI –0.33 to 0.17), with no evidence of heterogeneity (I² = 0%). This suggests that butyrate concentrations did not differ significantly between IBS patients and HCs across the included studies. For propionate (Fig. 2D), five studies- Tana 2010, Halmos 2014, Farup 2016, Fukui 2020, and Shin 2024- contributed data, with 204 IBS patients and 100 controls. The pooled SMD was 0.63 (95% CI 0.11–1.15), indicating significantly higher propionate concentrations in IBS patients compared to HCs (Z = 2.39, *p* = 0.017). However, heterogeneity was considerable (I² = 69.2%), reflecting variability across studies, particularly the large effect observed in Shin 2024.

Six studies contributed data on fecal SCFA concentrations in IBS-D compared to healthy controls.

For total fecal SCFAs (Fig. 3A), four studies- Sun 2021, Ringel Kulka 2015, Sagar 2020, and Waseem 2023- including 285 IBS-D patients and 159 controls were analyzed. The pooled SMD was 0.09 (95% CI –0.11 to 0.29), with low heterogeneity (I² = 12%), indicating no significant difference in overall fecal SCFA concentrations between IBS-D patients and controls. For acetate (Fig. 3B), six studies- Sun 2021, Tian 2020, Fukui 2020, Ringel Kulka 2015, Sagar 2020, and Waseem 2023- contributed data from 339 IBS-D patients and 199 controls. The pooled SMD was 0.03 (95% CI –0.29 to 0.34), with substantial heterogeneity (I² = 60.4%). While Fukui 2020 reported significantly lower acetate in IBS-D patients and Waseem 2023 observed higher levels, other studies showed no consistent differences, resulting in an overall non-significant effect. For butyrate (Fig. 3C), all six studies- Sun 2021, Tian 2020, Fukui 2020, Ringel Kulka 2015, Sagar 2020, and Waseem 2023- contributed data from 339 IBS-D patients and 199 controls. The pooled SMD was –0.12 (95% CI –0.37 to 0.12), with moderate heterogeneity (I² = 38.2%). While Sun 2021 and Fukui 2020 reported lower butyrate in IBS-D patients, other studies found no significant differences, resulting in an overall non-significant effect. For propionate (Fig. 3D), six studies- Sun 2021, Tian 2020, Fukui 2020, Ringel Kulka 2015, Sagar 2020, and Waseem 2023- including 339 IBS-D patients and 199 controls were analyzed. The pooled SMD was 0.30 (95% CI 0.10–0.49), indicating significantly higher propionate concentrations in IBS-D patients compared to controls (Z = 3.01, *p* = 0.003). Heterogeneity was negligible (I² = 0%), suggesting consistency across studies.

Four studies contributed data on fecal SCFA concentrations in IBS-C compared to healthy controls.

For total SCFAs (Fig. 4), three studies- Chassard 2012, Ringel Kulka 2015, and Waseem 2023- including 51 IBS-C patients and 58 controls were analyzed. The pooled SMD was –0.56 (95% CI –0.94 to –0.17), indicating significantly lower total fecal SCFA concentrations in IBS-C patients compared to healthy controls (Z = –2.80, *p* = 0.005). Heterogeneity was negligible (I² = 0%), suggesting consistency across studies. For acetate (Fig. 4B), four studies- Chassard 2012, Fukui 2020, Ringel Kulka 2015, and Waseem 2023- contributed data from 78 IBS-C patients and 84 controls. The pooled SMD was –0.06 (95% CI –0.46 to 0.34), with moderate heterogeneity (I² = 29.9%). Overall, acetate concentrations did not differ significantly between IBS-C patients and controls. For butyrate (Fig. 4C), four studies- Chassard 2012, Fukui 2020, Ringel Kulka 2015, and Waseem 2023- contributed data from 78 IBS-C patients and 84 controls. The pooled SMD was –0.22 (95% CI –0.57 to 0.14), with moderate heterogeneity (I² = 41.4%). This suggested no significant difference in butyrate concentrations between IBS-C patients and controls. For propionate (Fig. 4D), four studies- Chassard 2012, Fukui 2020, Ringel Kulka 2015, and Waseem 2023- including 78 IBS-C patients and 84 controls were analyzed. The pooled SMD was –0.22 (95% CI –0.98 to 0.54), with substantial heterogeneity (I² = 81%). Results were inconsistent, with some studies reporting lower propionate and others higher, leading to an overall non-significant effect.

Discussion

This meta-analysis provides important insights into SCFA profiles in IBS and its subtypes. Across all cohort studies, propionate concentrations were consistently elevated in IBS patients and particularly in IBS-D patients, whereas acetate and butyrate showed no significant differences compared to healthy controls. In IBS-C patients, total SCFA concentrations were significantly reduced compared to healthy controls, with consistent findings across studies and negligible heterogeneity. In contrast, individual metabolites (acetate, propionate, and butyrate) did not show significant pooled differences, and heterogeneity was moderate to high, reflecting variability across cohorts.

Recent systematic reviews also corroborate this study’s findings. Ju *et al*. [4] reported that propionate was the most consistently elevated SCFA in IBS patients, linking it to altered microbial fermentation and visceral hypersensitivity. Similarly, Gargari *et al*. [18] demonstrated that fecal SCFA profiles, particularly propionate, could stratify non-constipated IBS patients into clinically relevant subgroups. These observations support the role of propionate as a potential biomarker for IBS, especially diarrhea-predominant cases. Moreover, Shin *et al*. [17] highlighted that SCFA-microbiota relationships do underlie clinical heterogeneity in IBS.

The elevated levels of propionate identified in the present meta-analysis may mechanistically contribute to IBS pathophysiology through several pathways. Propionate can stimulate enteroendocrine cells to release serotonin, thereby accelerating gut motility and exacerbating diarrhea in IBS-D [19–21]. It also activates nociceptive pathways, enhancing visceral hypersensitivity [22, 23]. Furthermore, propionate has immunomodulatory effects, including promotion of Th17 responses, which may contribute to low-grade mucosal inflammation in IBS [24, 25]. These mechanisms provide a biological rationale for the symptomatology observed in IBS subtypes. Although SCFAs are generally considered beneficial for gut health, their physiological effects are concentration-dependent, and excessive production may contribute to gastrointestinal dysfunction [26]. In particular, increased abundance of propionate-producing bacteria such as *Akkermansia* and *Veillonella* has been implicated in disease severity under specific pathological conditions. Shin *et al*. [17] also suggested that microbial signatures characterized by *Prevotella copri*-associated propionate production and expansion of *Akkermansia* may play a significant role in the pathogenesis of IBS-C. Furthermore, *P. copri* has been linked with inflammatory conditions, including subclinical and new-onset rheumatoid arthritis, indicating its potential pro-inflammatory role beyond the gastrointestinal tract [27]. Additionally, bacteria belonging to the genus *Prevotella* possess mucin-degrading enzymatic activity, which may compromise mucus layer integrity, alter epithelial barrier function, and potentially contribute to increased intestinal permeability [28]. Thus, the recent and emerging evidence on the SCFA-microbiota relationship highlight the need to consider subtype-specific metabolic signatures.

The heterogeneity observed in acetate and butyrate analyses is noteworthy. Moderate to substantial variability (I² ranging from 38% - 83%) suggests that dietary intake, microbiome composition, and methodological differences across studies strongly influence SCFA measurements. In particular, heterogeneity in propionate outcomes was substantial in the overall IBS cohort and IBS-C subgroup. This may reflect differences in dietary fiber intake, stool consistency, and analytical methods (gas chromatography vs. NMR spectroscopy). Patient demographics, including sex-based differences and geographic dietary patterns, further contribute to variability. In IBS-C, where total SCFAs were consistently reduced but individual metabolites showed divergent results, heterogeneity may reflect differences in fiber intake and stool consistency, both of which are highly variable across populations. This variability underscores the importance of conducting region-specific studies, as dietary patterns and microbiome composition in India differ substantially from Western cohorts.

To strengthen methodological rigor, we conducted a NOS quality assessment of the included studies. The traffic-light plot (Fig. 1, Supplementary material) demonstrated that most studies were of good quality (score ≥6), with consistently low risk in the outcome domain. Variability was primarily observed in the comparability domain, where several studies had only partial adjustment for confounding factors such as diet, BMI, age, and sex. Only one study (Farup 2016) was rated as moderate quality (score of 5), while none were classified as poor quality. This assessment increases confidence in the robustness of the pooled findings.

In India, IBS prevalence is estimated at 4–7% of the adult population, with subtype distribution showing IBS-D predominance in urban populations and IBS-C more common in rural cohorts [6, 29]. Indian consensus guidelines emphasize the heterogeneity of IBS presentations and the need for culturally adapted diagnostic approaches [29]. Given this epidemiological context, the consistent elevation of propionate in IBS-D observed in our meta-analysis could be particularly relevant for Indian patients, where diarrhea-predominant IBS is the dominant subtype in metropolitan areas. Incorporating fecal or serum propionate measurement into diagnostic frameworks may provide an objective biomarker, complementing symptom-based Rome IV criteria that are often limited by cultural perceptions of bowel habits and underreporting. Conversely, the significant reduction in total SCFAs in IBS-C highlights a distinct metabolic signature that may be clinically useful for identifying constipation-predominant cases, especially in rural Indian populations.

While SCFA biomarkers hold promise, their clinical feasibility must be considered. Fecal SCFA analysis typically requires gas chromatography or NMR spectroscopy, which are available in tertiary centers but remain costly. Standardization of sample collection and storage is essential for reproducibility. Serum SCFA assays are less widely available but may offer greater clinical applicability. Future work is required to evaluate cost-effectiveness and accessibility in routine practice, particularly in resource-limited settings.

A key limitation of this meta-analysis is that most included studies were conducted in Western or East Asian populations, with no Indian cohorts represented. This restricts the generalizability of findings to the Indian context, where dietary diversity, microbiome composition, and environmental exposures differ substantially. Given that SCFA production is highly dependent on fiber intake, fermentation substrates, and gut microbial ecology, extrapolating results from non-Indian cohorts may overlook region-specific metabolic signatures. Another limitation is the heterogeneity observed across studies, particularly for acetate, propionate, and butyrate in IBS-C. Variability in study design, analytical methods (*e.g.*, gas chromatography vs. NMR spectroscopy), and sample handling likely contributed to inconsistent findings. In the Indian context, heterogeneity may be further amplified by diverse dietary practices (*e.g.*, vegetarian vs. mixed diets, regional staples such as rice vs. wheat), antibiotic exposure, and cultural differences in symptom reporting. This underscores the need for standardized protocols and larger, multicenter studies in India to reduce variability and improve reproducibility.

Future research should prioritize subtype-specific SCFA profiling in Indian IBS cohorts, integrating fecal metabolomics with microbiome sequencing to capture both microbial composition and functional output. Such studies could clarify whether propionate elevation in IBS-D is reproducible in Indian populations and whether reductions in total SCFAs in IBS-C are consistent across dietary and regional contexts. Additionally, longitudinal studies assessing SCFA dynamics in response to dietary interventions (*e.g.*, low-FODMAP diets, probiotic supplementation) could provide mechanistic insights and guide personalized therapy.

From a translational perspective, SCFA biomarkers hold promise for enhancing IBS diagnosis and management in India. Incorporating fecal or serum propionate measurement into clinical practice could complement Rome IV criteria, reduce misclassification, and enable targeted interventions. Given the rising burden of functional gastrointestinal disorders in urban India, biomarker-based stratification may improve early diagnosis, optimize dietary counseling, and support precision medicine approaches. Ultimately, validating SCFA signatures in Indian cohorts will be critical for bridging global evidence with local clinical practice.

## Supplemental Materials

Supplementary data for this paper are available on-line only at http://jmb.or.kr.



## Figures and Tables

**Fig. 1:**
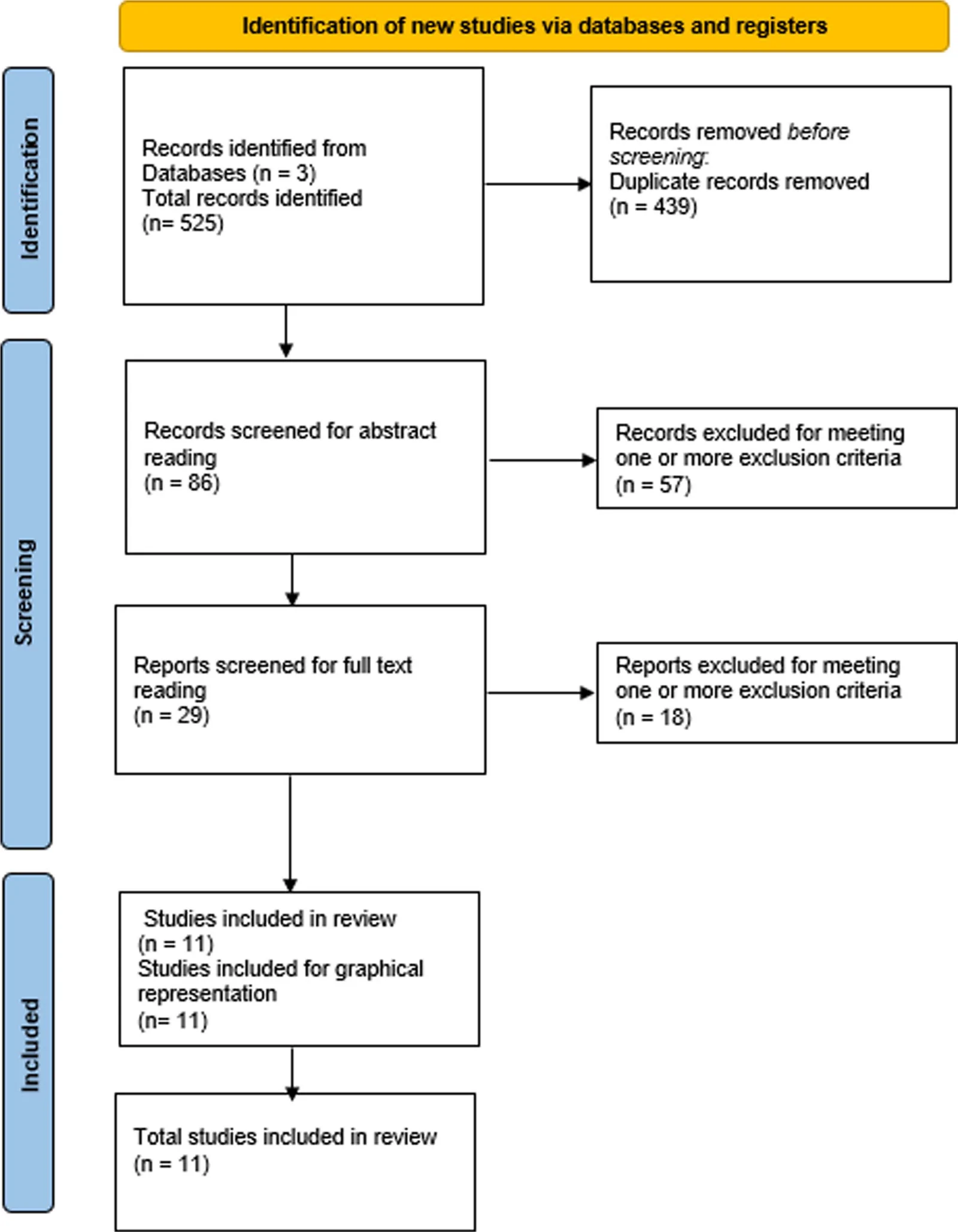
The PRISMA flowchart for selection of studies.

**Fig. 2:**
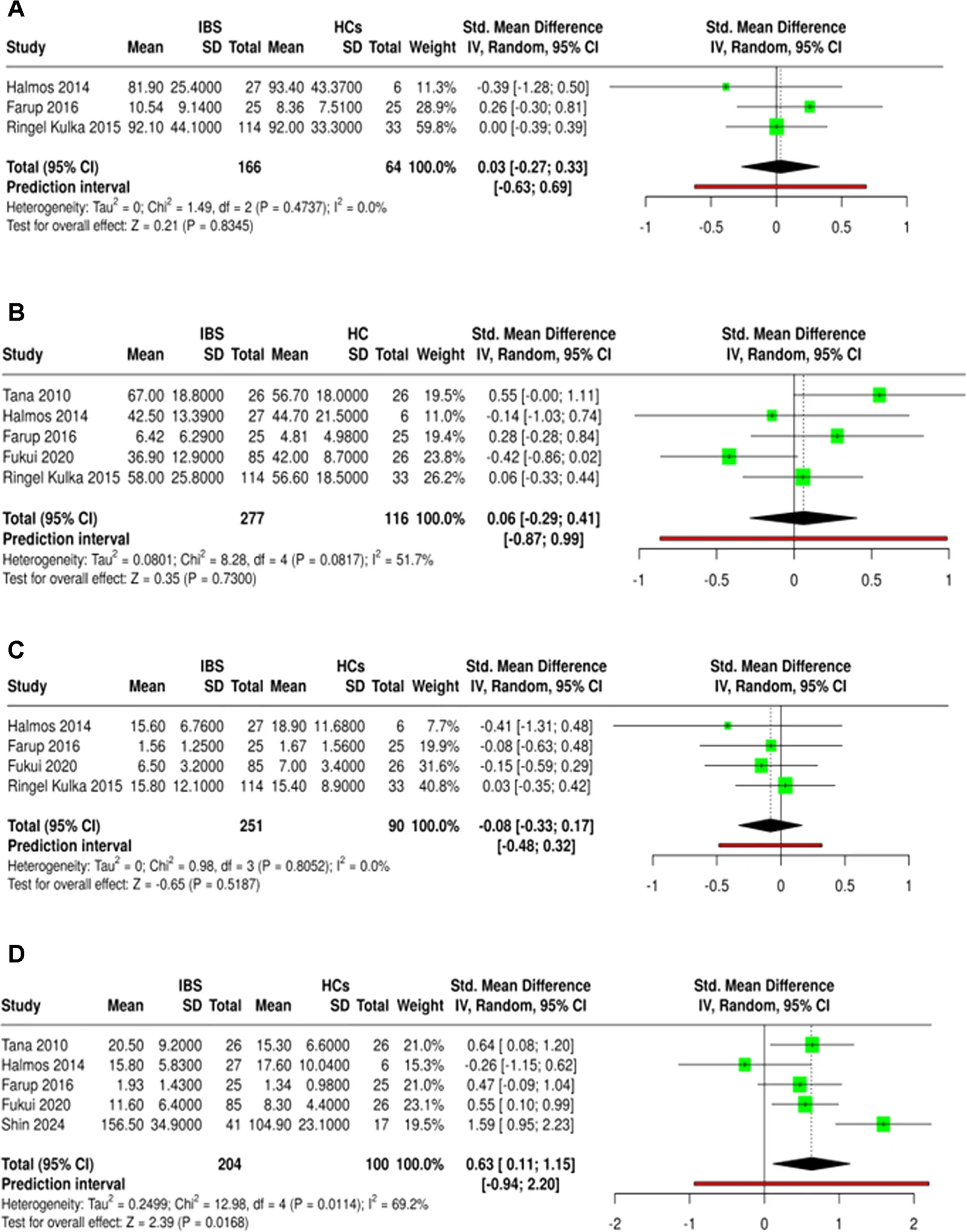
Forest plots of alterations of concentration of fecal SCFAs in IBS patients versus HCs. (**A**) total SCFAs, (**B**) acetate, (**C**) butyrate, (**D**) propionate.

**Fig. 3:**
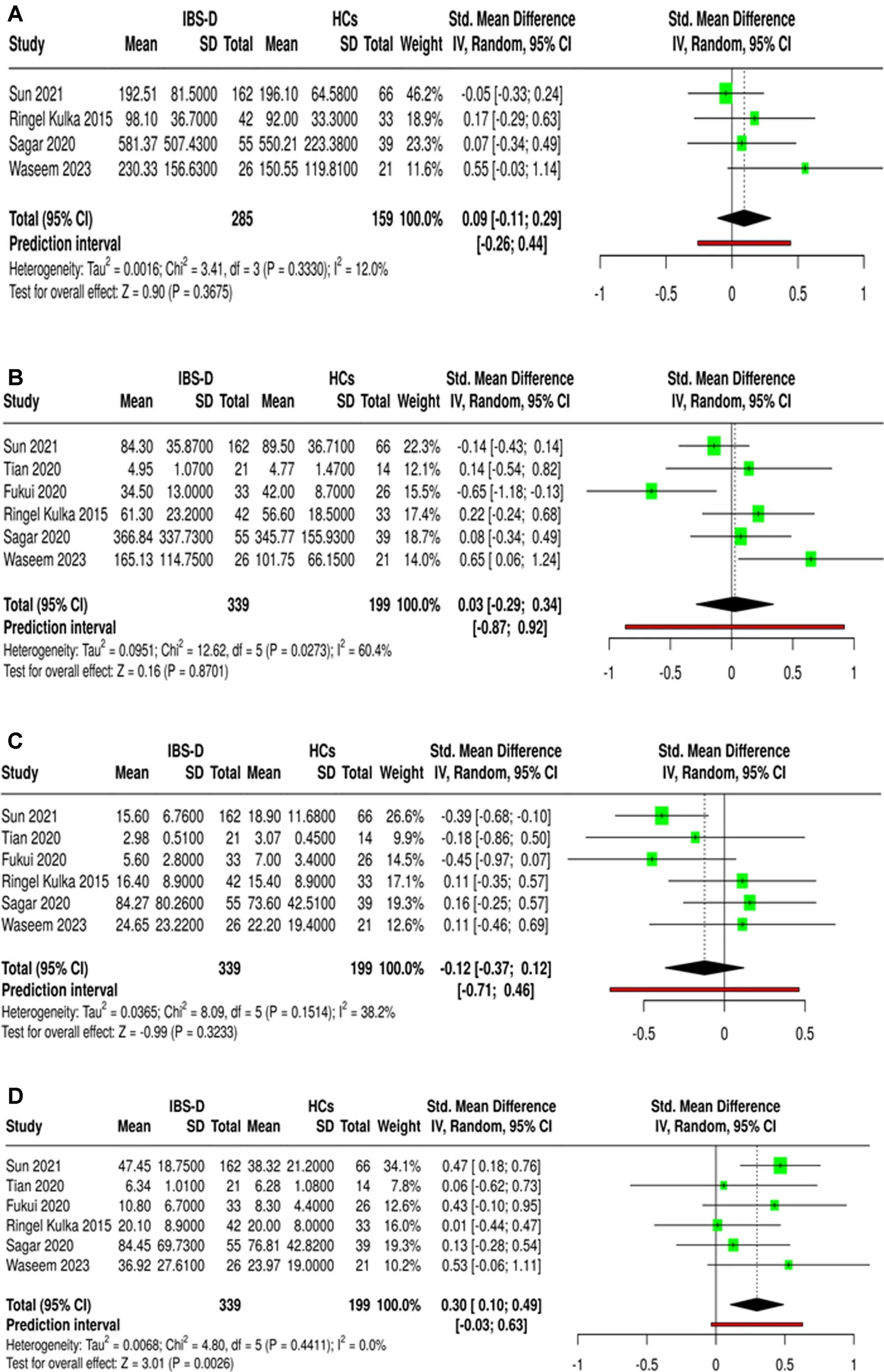
Forest plots of alterations of concentration of fecal SCFAs in IBS-D patients versus HCs. (**A**) total SCFAs, (**B**) acetate, (**C**) butyrate, (**D**) propionate.

**Fig. 4:**
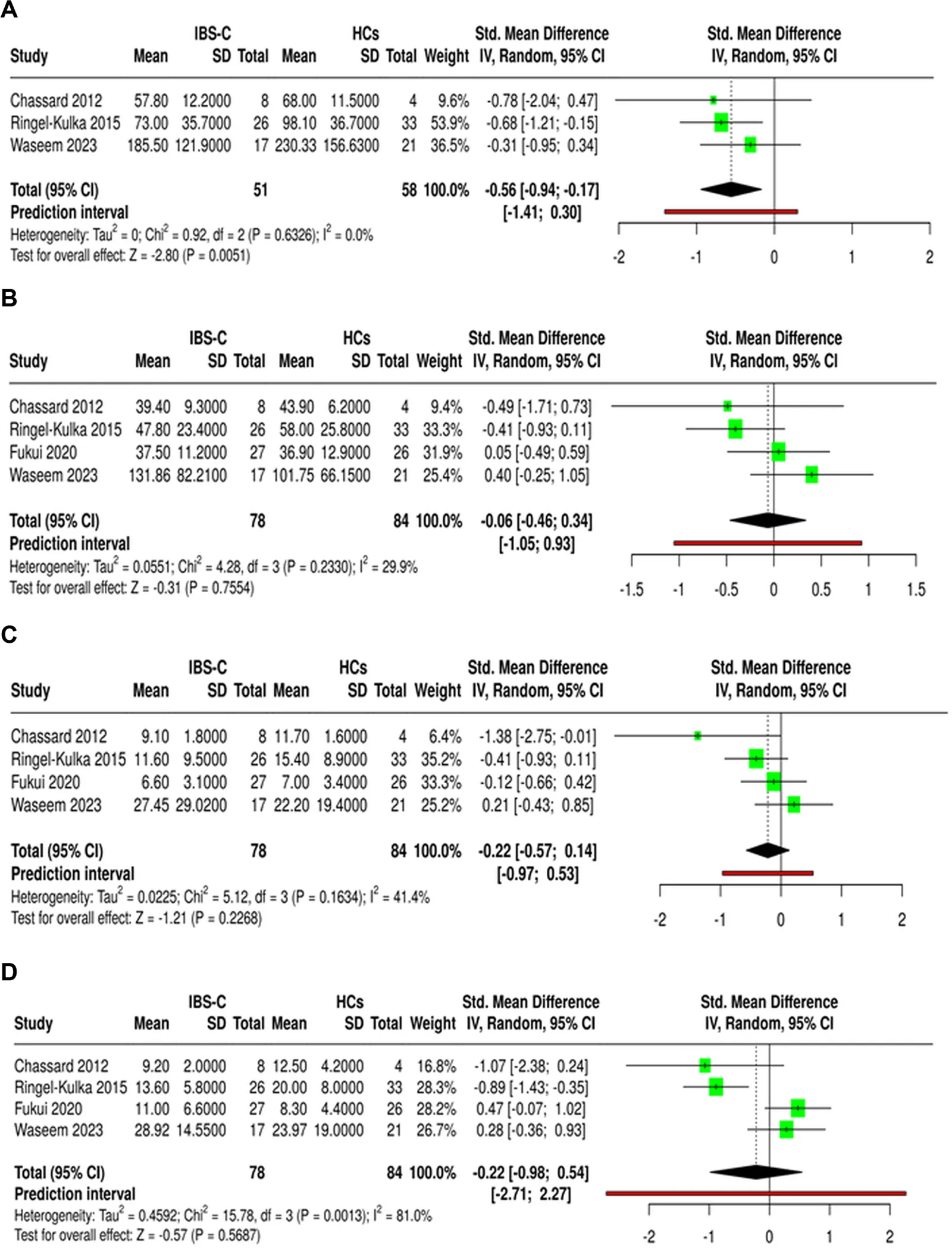
Forest plots of alterations of concentration of fecal SCFAs in IBS-C patients versus HCs. (**A**) total SCFAs, (**B**) acetate, (**C**) butyrate, (**D**) propionate.
