# Supplementary material for: SCFA Alterations in IBS Subtypes: Systematic Evidence and Future Prospects for Biomarker-Based Diagnosis in India
Source: J Microbiol Biotechnol. 2026 Sep 2;36:e2605029. doi: 10.4014/jmb.2605.05029 (PMC13655589; doi:10.4014/jmb.2605.05029)
Supplement: Supplementary file 1 [file jmb-36-e2605029-supple.pdf]

J. Microbiol. Biotechnol. 2026. 36: e2605029

https://doi.org/10.4014/jmb.2605.05029

Supplementary Tables and Figure

Table S1. Characteristics of studies included.

Studies included	Year	Country	Diagnostic criteria followed	IBS/ HC	IBS-Age (Mean)	HCs-Age (Mean)	IBS-Sex (M/F)	HCs-Sex (M/F)	Format of reported data	Method of SCFA analysis
Tana	2010	Japan	Rome II	26/26	21.7	21.9	13/13	13/13	Mean, SD	HPLC
Chassard	2012	France	Rome II	14/12	48	30	0/14	0/12	Mean, SD	1H NMR
Halmos	2015	Australia	Rome III	27/6	41.9	39.4	6/21	1/5	Mean, 95% CI	GC
Ringel-Kulka	2015	USA	Rome III	114/33	35.4	33.9	16/98	4/29	Mean, SD	GC
Farup	2016	Norway	Rome III	25/25	46.2	49.2	12/13	10/15	Mean, SD	GC
Fukui	2020	Japan	Rome III	85/26	51.3	46.2	37/48	9/17	Mean, SD	GC-MS
Sagar	2020	UK	Rome III	20/26	55.4	55.4	4/14	11/15	Median, IQR	GC
Tian	2020	China	Rome IV	21/14	31.6	27	13/8	8/6	Mean, SD	HS-SPME-GC-MS
Sun	2021	China	Rome III	162/66	35.5	36.7	114/48	38/28	Median, IQR	UPLC-MS/MS
Waseem	2023	USA	Rome IV	43/21	Data not available	32.1	10/33	5/16	Median, IQR	HPLC
Shin	2024	USA	Rome IV	41/17	35.5	31.7	10/31	12/5	Median, IQR	HPLC-MS

Table S2. NOS domain ratings and total scores for included studies.

Study	Selection	Comparability	Outcome	Total score
Tana 2010	Low (3)	Some concerns (1)	Low (2)	6
Chassard 2012	Low (3)	Some concerns (1)	Low (2)	6
Halmos 2014	Low (4)	Low (2)	Low (3)	9
Ringel-Kulka 2015	Low (3)	Some concerns (1)	Low (2)	6
Farup 2016	Some concerns (2)	Some concerns (1)	Low (2)	5
Fukui 2020	Low (3)	Some concerns (1)	Low (2)	6
Sagar 2020	Low (3)	Some concerns (1)	Low (2)	6
Tian 2020	Low (3)	Low (2)	Low (2)	7
Sun 2021	Low (3)	Low (2)	Low (2)	7
Waseem 2023	Low (3)	Low (2)	Low (2)	7
Shin 2024	Low (3)	Low (2)	Low (2)	7

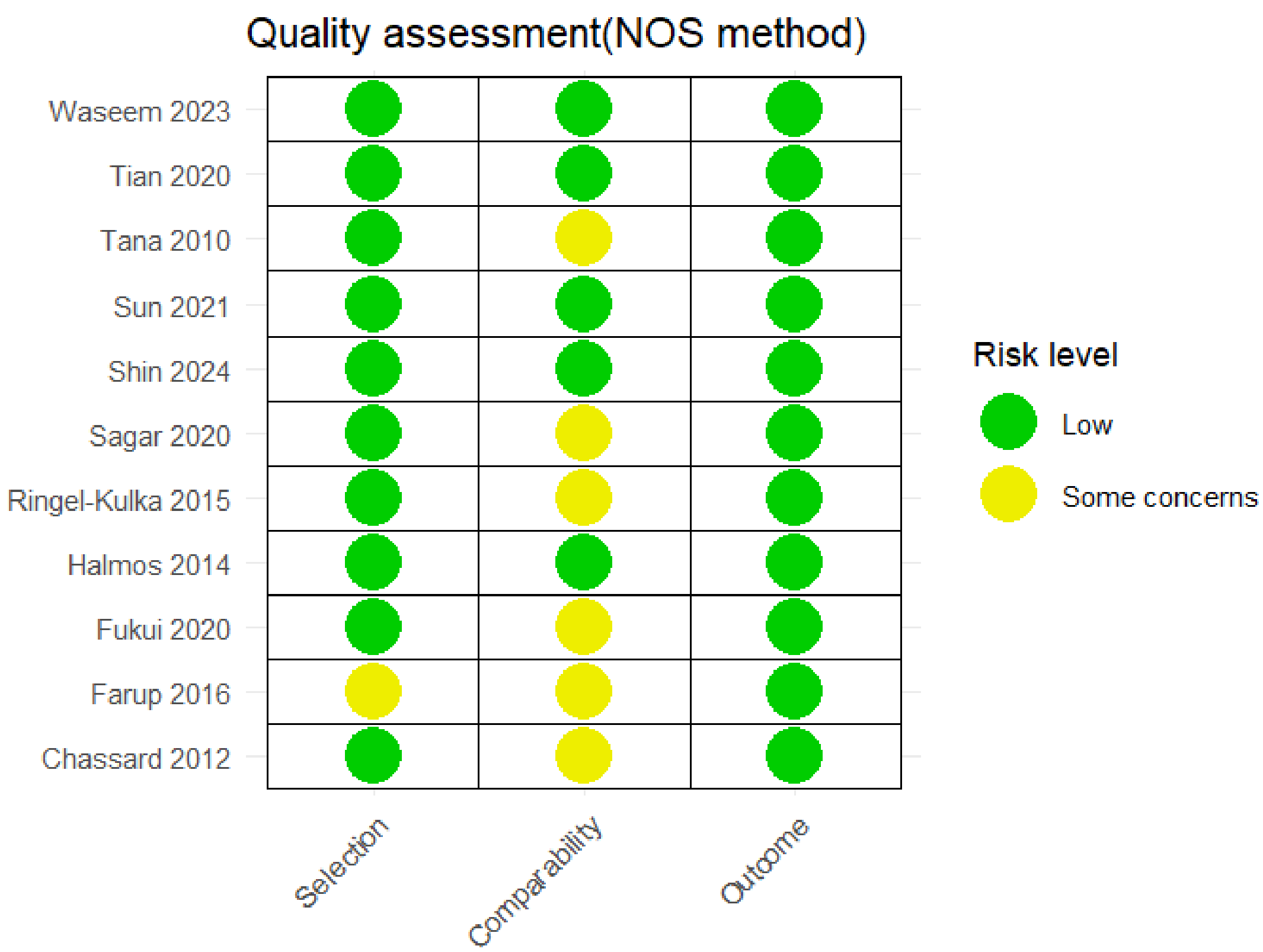

Fig. S1. Traffic light plot of methodological quality assessment using the Newcastle–Ottawa Scale (NOS).
